# Interventional and Non-interventional Medical Rehabilitation Approaches to Axial Spine Pain in Vertebral Metastatic Disease

**DOI:** 10.3389/fpain.2021.675787

**Published:** 2021-06-04

**Authors:** Krishna Sarma, David J. Kohns, Maryam A. Berri, Elizabeth Joyce, Sean R. Smith

**Affiliations:** ^1^Department of Physical Medicine and Rehabilitation, University of Michigan Medical School, Ann Arbor, MI, United States; ^2^University of Michigan Medical School, Ann Arbor, MI, United States

**Keywords:** cancer pain, cancer rehabilitation, spinal metastases, back pain, injection safety

## Abstract

As targeted therapies help patients with advanced cancer live longer, interventions for management of axial spine pain will become more common. Unfortunately, the indications for and safety of these procedures have been relatively unexplored compared with non-cancer adults. This review focuses on the following aspects of axial spine pain management in patients with vertebral metastatic disease: (1) pathophysiology and symptoms of cancer- and non-cancer-related spine pain; (2) safety and efficacy of non-interventional rehabilitation approaches to treat this pain; (3) considerations for interventional pain approaches to acute and chronic pain in patients with vertebral metastatic disease. This review also summarizes gaps in the literature and describes specific cases in which the described interventions have been applied.

## Introduction

The prevalence of vertebral metastatic disease (VMD) continues to increase due to improved treatment options of primary tumors that give rise to vertebral metastases [1]. Approximately two-thirds of adults with cancer that have metastasized to bone will develop VMD, although not all patients will have pain nor neurologic deficits [2]. Conversely, non-malignant back pain is incredibly common −80% of all adults will develop non-cancer low back pain (LBP) in their lifetime—making identifying the pain generator in patients with VMD and LBP both challenging and critical, as the treatment for each is drastically different [3]. Management of pain in VMD requires a multidisciplinary framework, including interventional procedures and non-interventional rehabilitation approaches when cancer is not generating the pain [4]. Unfortunately, it is not always clear what interventions can be safely and effectively administered to patients with VMD. The presence of the tumor, as well as a patient's current and prior treatment, and laboratory parameters, including platelet and absolute neutrophil count (ANC), all confound decision-making. This review summarizes rehabilitation approaches to diagnosing and managing LBP in patients with VMD.

## Differentiating Malignant and Non-Malignant Back Pain

Back pain in the setting of VMD can be generated by the malignancy itself or from more typical pain generators—herniated disks, spondylosis, muscle strain, and more. In fact, the presence of VMD may predispose patients to developing mechanical, non-cancer LBP due to cachexia and focal myopathies that can develop with pro-inflammatory cytokine release and cancer-related malnutrition [5]. Muscular strain is the most common cause of non-malignant back pain; however, mechanosensitive and chemosensitive nociceptor activation, following trauma and altered loading on the vertebral column can also cause non-malignant back pain, as observed in spondylosis [6]. Therefore, if the spinal tumor is not causing neurologic compromise or pain, other pain generators must be considered. Physical exam findings would be typical of non-malignant back pain, and imaging studies may show chronic or acute changes consistent with non-cancer pain generators (e.g., disc herniation causing narrowing of the lateral recess). A summary of all non-cancer pain generators that cause back pain are out of the scope of this focused review, but the authors recommend Chiodo et al. for further reading [7].

A thorough history and physical examination in conjunction with diagnostic imaging studies are all critical to discerning metastatic disease pain from common non-cancer musculoskeletal pain generators. Frequently, however, a patient's history may be congruent with his or her physical exam while incongruent to imaging studies. There have been cases of otherwise healthy individuals referred to a “specialty clinic” with a complaint of back pain that is later determined to be an undiagnosed cancer; to wit, Zaikova et al. reported that, though rare, the prevalence of metastatic spinal cord compression is higher than expected at the time of cancer diagnosis [8]. Given the limitations of current diagnostic imaging, a back pain diagnosis in the setting of malignancy is an evolving process that benefits from thoughtful analysis of a patient's response to treatment.

## Malignant Back Pain: Types, Locations, and Quality

Malignant back pain may be due to either primary or secondary tumors (VMD), the latter of which are far more common than primary vertebral tumors in adults [9]. Vertebral metastatic disease is most commonly associated with lung, breast, prostate, renal, and thyroid carcinomas [10], with a recent large-scale retrospective study spanning 2007–2019, finding that lung cancer was the most common primary tumor, leading to VMD (36% of men and 37% of women) [11]. Pain related to malignancy is often unrelenting and is felt through effects on all structures of the spinal column. Tumors may compress neural structures, causing referred dermatomal pain or weakness. Pain may also be from the invasion of bony structures, causing nociceptive pain, and pro-inflammatory cytokines may further exacerbate inflammation and pain. In response to tumor cell invasion, the inflammatory cascade releases proteases, endothelin, tumor necrosis factor-α, serotonin, prostaglandins, and nerve growth factor, which excite bone-innervating sensory nerve endings. This leads to a web of continued growth, amplifying the progressive nature of malignant spine pain that may not cause focal neurologic deficits [12, 13]. It is important to note that cancer pain is often “multimorphic,” meaning that it is a dynamic process, multicomponent (i.e., neuropathic and nociceptive), and it may exacerbate or be exacerbated by other pain generators [14].

Tumors of the spinal column may be bony, epidural, intradural, or leptomeningeal, with each exhibiting characteristic clinical and radiological findings:

- *Bony tumors* cause pain from local invasion and from fractures that potentially lead to neurologic compromise. Computerized tomography (CT) or magnetic resonance imaging (MRI) can detect these, with plain radiographs being less sensitive. Bone scintigraphy may detect osseous disease but does not characterize the location of the tumor in the spinal column (e.g., anterior or posterior), and specificity is reduced due to potential false positives from degenerative changes or non-cancer fractures. A physical exam may be unremarkable if a bony tumor is not causing pain, but painful tumors will often be provoked by weight-bearing, loading/moving the spine, and, potentially, palpation. A thorough exam to evaluate for radicular symptoms is essential to determine if neurologic compromise is present.
- *Epidural tumors* tend to cause neuropathic symptoms (including pain) localizable to regional dermatomes/myotomes and may cause weakness below the level of the lesion if the spinal cord is affected. MRI is the preferred imaging choice when this is suspected. The physical exam may be similar to that of a patient with bony disease, as patients often have osseous involvement as well, although weakness and numbness may be more pronounced.
- *Leptomeningeal disease* represents a diffuse process that generally portends an unfavorable prognosis; patients may have widespread pain, numbness, and tingling associated with weakness, headaches, and bowel/bladder sensory loss incontinence. MRI may detect this and lumbar puncture would provide a definitive diagnosis and an aide in characterizing the tumor cells. The physical exam may be non-focal but often will be associated with multiple neurologic deficits.
- *Intradural disease* is relatively rare and often due to a primary tumor rather than metastasis. This process can cause symptoms of incomplete or complete spinal cord injury. MRI is the preferred diagnostic study, and the physical exam will vary, depending on the location of the tumor and the extent of spinal cord involvement.

## Considerations for Interventional Approaches to Malignant Spine Pain

For patients with known spinal malignancy, there are a number of safety measures to consider in performing an interventional spine procedure. As part of the informed consent, the patient should be made aware of potential risks, benefits, alternatives, and the possibility that the procedure will be discontinued if there appear to be abnormalities. A review of all available imaging is essential to ensure safe passage along the trajectory and the final target of the planned procedure, as well as a detailed discussion of altered anatomy and the plans to accommodate these structural changes.

Evaluating the nature of the spinal tumor, including its location and risk of neurologic compromise, is crucial when determining potential interventions. The spine instability neoplastic score (SINS) is a framework by which providers can estimate the stability of VMD to determine if a lesion can be treated conservatively or if it requires urgent intervention with radiation and/or surgery [15]. This schema characterizes tumors based on location, a type (e.g., lytic or blastic), how much of the vertebral body is compromised, presence of pain, and more. Essentially, tumors, which occupy a substantial portion and/or the posterior aspect of the vertebral body, are in a relatively mobile segment of the spine (cervical or lumbar), or which cause pain, and are more likely to result in neurologic compromise. Tumors that are smaller, anterior, blastic, and located in less-mobile spine segments are less likely to result in instability, necessitating emergent care. The standard of care for malignant spinal cord compression—of which only an estimated 10% of patients with VMD will develop—is surgical decompression as soon as possible to prevent irreversible neurological compromise from spinal cord injury, followed by radiation therapy [16]. Therefore, it is important to document a baseline neurologic examination just before the procedure to identify any neurologic compromise.

**Figure 1:**
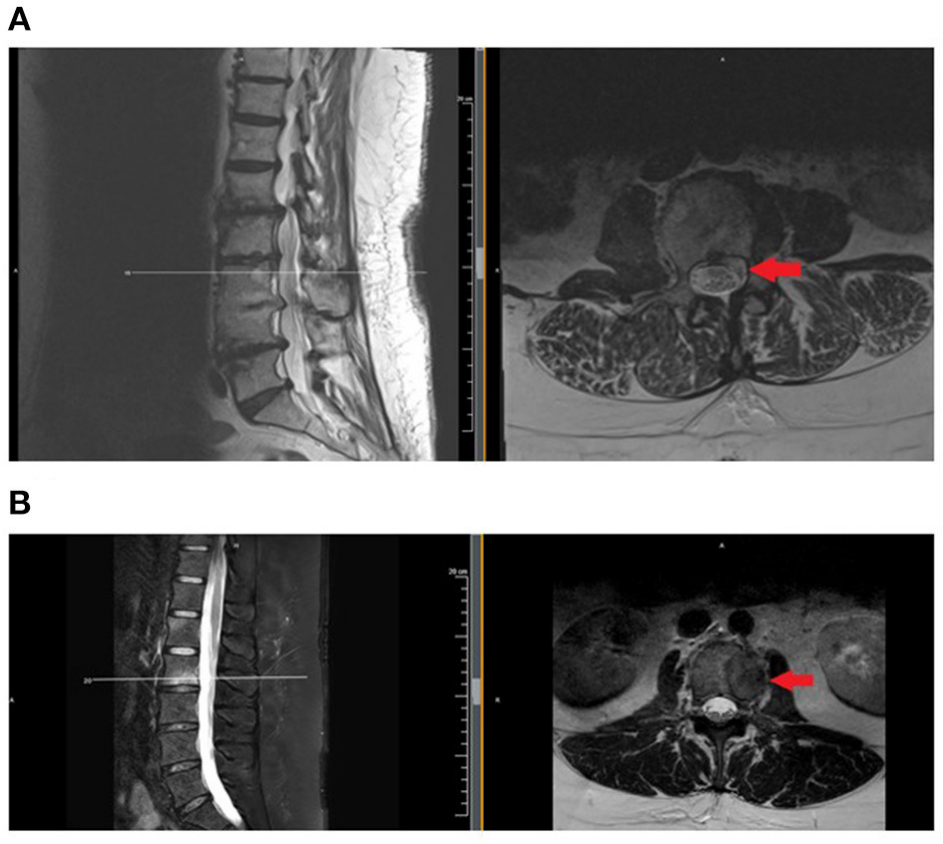
Examples of patients with vertebral metastatic disease, involving the epidural space **(A)** and confined to osseous structures. **(B)** Injections should be avoided or administered with extreme caution when disease involves the epidural space. MRI is the test of choice to evaluate for epidural disease. MRI, magnetic resonance imaging.

Generally, contraindications for spinal procedures in malignancy are if there is a risk of seeding tumor cells into the spinal column, if the tumor burden is extra-osseous and in the expected path of a needle, and if there is radiologic or clinical evidence of spinal cord compression or myelopathy. For example, spine injections should not be administered at a level that contains epidural tumor, as they risk seeding the tumor and/or causing bleeding, as tumors tend to be highly vascularized. Figure 1 shows two axial MRI cuts of patients with VMD: one in the epidural space and one with disease localized to the vertebral body and, therefore, out of a needle's path if performing a transforaminal epidural steroid injection.

Patients with cancer are at risk of thrombocytopenia and/or neutropenia, which can increase the risk of bleeding or infection, respectively, and, also, must be considered when planning an interventional procedure. Patients with hematologic malignancies, or solid tumors receiving new cytotoxic chemotherapy, are at the biggest risk [17]. As a hematoma or infection near the spine can be catastrophic, patients with active or recently treated cancer should have a complete blood count checked within 2–3 weeks of an interventional procedure. The safety of these procedures while patients are receiving cytotoxic chemotherapy has not been definitively proved; however, a recent retrospective analysis at a single tertiary care medical center cancer rehabilitation program suggests that if neutrophil and platelet counts reach a threshold of 1.5 K/μL and 100 K/μL or greater, respectively, there may not be an increased risk of adverse events [18]. In a recent study, there has been level III evidence for epidural steroid injections to treat pain related to spinal malignancy; specifically, the thoracic and lumbosacral regions demonstrated greater efficacy relative to injections in the caudal spine [19].

Additionally, certain anticancer therapies may contraindicate interventional procedures. Bevacizumab, which inhibits vascular endothelial growth factor and is used to treat tumor-related edema and slow progression in some malignancies, increases the risk of bleeding. As the medication has a half-life of 18–21 days, an interventional procedure may need to be planned around the patient's bevacizumab infusion schedule, which is typically every 2–3 weeks [20]. Immunotherapy, which has become a mainstay of the treatment of advanced solid tumors, relies on the body's innate immune response to target malignant cells. Therefore, a procedure that administers a steroid—and, therefore, potentially blunts the body's immune system response—must be considered only after discussion with the patient's medical oncologist and the patient's understanding that there is a theoretical but, by no means, proven risk of reducing the efficacy of the immunotherapy.

**Figure 2:**
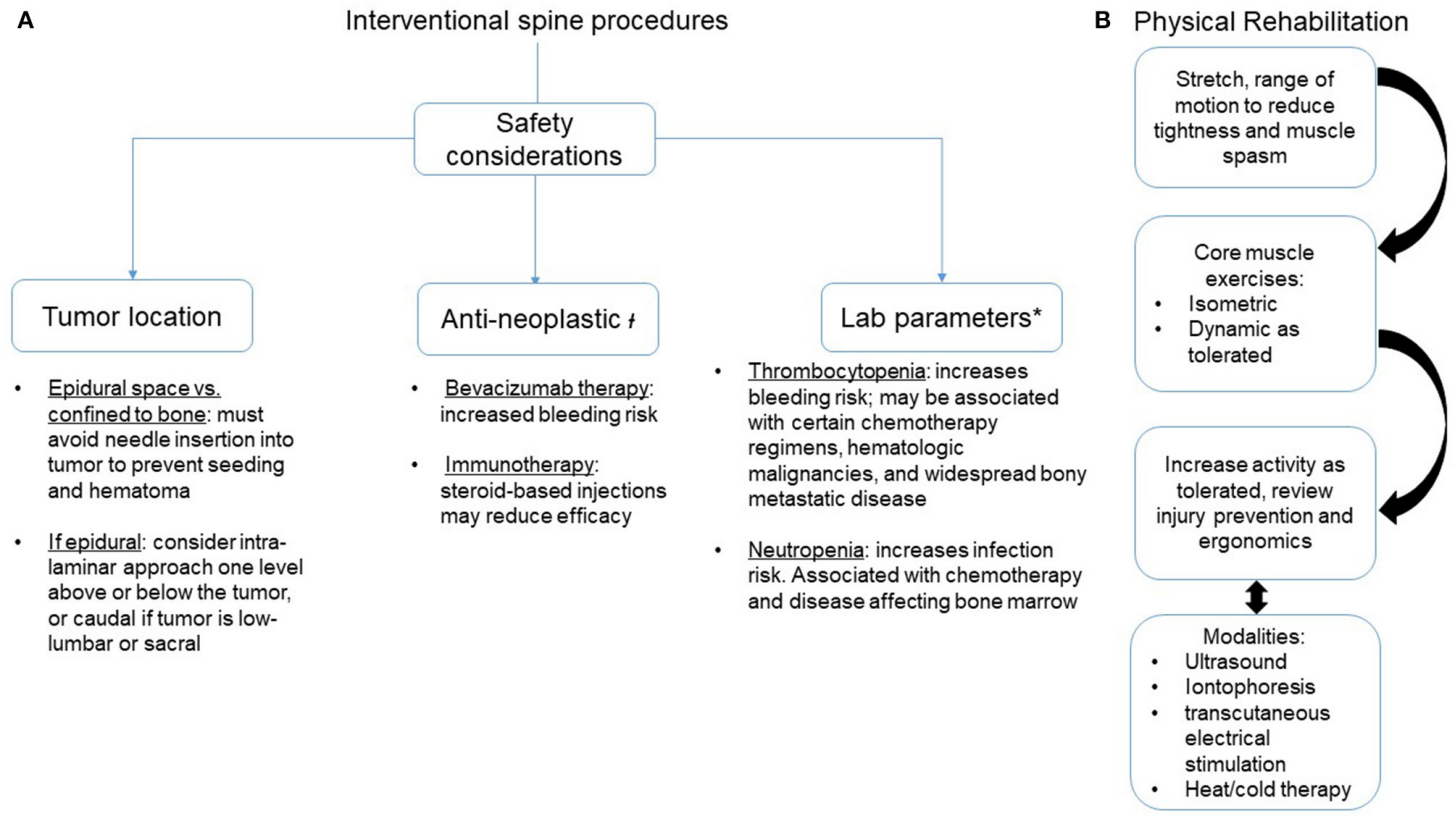
Interventional spine procedures and rehabilitation strategies to address axial spine pain in patients with vertebral metastatic disease. **(A)** Safety considerations. **(B)** Sequence of rehabilitation methods. ł, Recommend discussing with the oncology team before proceeding; *, No agreed-upon standards for safe levels. Recommend transfusing platelets if below 50,000 units and considering if under 100,000 units. Recommend a granulocyte colony stimulating factor for an absolute neutrophil count below institutional safe thresholds, often < 1.5 units.

After taking into consideration safety measures, contraindications, radiographic studies, anatomical variants, systemic therapy, and laboratory parameters (Figure 2), patients with spinal metastases may be offered certain interventional procedures to address their back pain. However, compression fractures in patients with VMD must also be critically evaluated before an interventional procedure. Patients with cancer may have compression fracture-related pain for different reasons: malignant pain is from disease, eroding the bony integrity of the vertebral body, or pain may be unrelated to a tumor and from insufficiency, as cancer patients are at higher risk of developing non-cancer compression fractures [21]. The etiology of a compression fracture can be determined by CT or MRI (to evaluate for tumor invasion), positron emission computerized tomography (PET-CT; to determine if there is heightened metabolic activity at the fracture site), and, definitively, via a biopsy. Cancer-related fractures should obviously be triaged for radiation and/or surgical care. For non-cancer vertebral compression fractures, acute fractures must be evaluated for spinal stability. If stable, orthotic bracing and oral analgesics are the first line for treatment. If the pain can be localized to the fracture level and oral analgesia is insufficient, a 2016 systematic review concluded that percutaneous vertebral augmentation (vertebroplasty or kyphoplasty) is safe and effective for the cancer-related vertebral compression fracture [22]. Chronic post-fracture pain can also develop, which is characterized by dull, aching pain that is worse with extension due to increased loading of facet joints around the fracture site and may be amenable to medial branch radiofrequency ablation (RFA).

Neurosurgical interventions, including cordotomy, cingulotomy, and intravertebral RFA of symptomatic lesions, should be considered for refractory cancer pain but are beyond the scope of this review. The decision to perform these procedures should not be made without multidisciplinary discussion with the members of the oncology team.

## Pharmacological and Rehabilitation Strategies to Address Benign and Malignant Spine Pain

There are four broad categories that are typically used in the approach for pain-control management. This is utilized in most conditions that generate pain, whether it be acute or chronic, neuropathic, central or nociceptive, focal or widespread. They include exercise and skilled therapies, medications, interventional procedures, and behavioral techniques (Figure 2). Numerous studies that look at chronic back pain have revealed the benefits of a multidisciplinary approach to pain management. This includes active therapies (strengthening, cardiovascular fitness, stretching, etc.) and behavior approaches (cognitive behavior therapy, mindfulness, progressive relaxation, etc.) [23, 24]. However, there can be hesitation from both the provider and the patient in initiating a rigorous rehabilitative program due to safety concerns and fear of exacerbation of painful stimulus. Thus, the balance between the risk and the benefit of such programs remains complicated. Clinicians should assess for safety by appropriately completing baseline musculoskeletal and neurological examinations that evaluate mechanical axial loading, signs of spinal cord and peripheral nerve compression, and change in sensorimotor and autonomic functioning. Additionally, the SINS criteria can be useful for evaluating a disease burden and to serve as a baseline for warning.

There are numerous pharmacological treatment options that can decrease the painful stimulus of tumor invasion and periosteal stretch in the vertebrae. Medication management in this setting includes a stepwise approach, consistent with World Health Organization (WHO) and European Society of Medical Oncology (ESMO) guidelines, starting with the anti-inflammatory cascade, dulling the interpretation of nociceptive nerve firing, and decreasing the impulses that contribute to neuropathic pain [25–27]. Non-steroidal anti-inflammatory drugs (NSAIDs) and acetaminophen are commonly first-line medication choices for mild to moderate-level cancer-related pain; however, NSAIDs must be used with caution and may be contraindicated in patients with thrombocytopenia, on concurrent anticoagulant medication, multiple myeloma, or renal failure. These analgesics can be offered as an alternative to lower the opioid dose burden [28]. Acetaminophen with the addition of codeine is also a reasonable option for moderate to severe cancer-related pain with further escalation reserved to medications such as hydrocodone, oxycodone, and other short- or long-acting morphine equivalents.

Approximately 15–40% of chronic cancer pain has a component of neuropathic pain; and tricyclic antidepressants (TCAs), antispasmodic agents, gabapentinoids, and antiepileptic medications are medications of choice for nerve-related pain, although their efficacy in managing bony cancer pain remains inconclusive [29–31] and may not be effective for discogenic (non-cancer) radicular pain [32]. In the absence of a bony component of cancer pain, gabapentin has been shown to be effective in managing neuropathic cancer pain [33]. Topical medications that utilize the above agents, including those compounded with an NSAID, should also be considered when a patient has superficial pain.

For bony tumor-related pain, opioid analgesia may be effective and is recommended by the WHO and the ESMO Oncology when cancer pain cannot be managed better with other analgesics. It is important to note that opioids produce more side effects than TCAs and gabapentin [34]. Patients who have opioid-responsive pain but cannot achieve optimal dosing due to side effects should be considered for an intrathecal opioid pump. Bisphosphonates are another medication management strategy that utilizes the potent inhibition of osteoclast-mediated bone resorption cells, decreasing the secretion of osteolytic cytokine-mediated inflammatory factors, but use of this medication should only be undertaken when discussed with the patient's medical oncologist since some anti-resorption medications are used for the treatment of bony metastatic disease.

In conjunction with pharmacological measures, exercise and physical and occupational therapy plays a critical role in treating spinal metastasis pain. Maintaining patients' mobility and facilitating their capacity to perform activities of daily living are vital components of a cancer rehabilitation program [35]. Rehabilitative therapies for axial spine cancer pain include manual stretching, myofascial therapy, passive mobilization, and active exercises as promotion of muscle strength and flexibility, improvement in aerobic capacity, and release of endogenous pain-relieving factors [36]. A personalized exercise plan, including skilled therapy, should be generated after a thorough review of a patient's neurologic and musculoskeletal physical exam and imaging studies to ensure safety. In the absence of neurologic compromise or an unstable lesion, therapy to strengthen core muscles appears to be safe in patients with VMD, even those that are actively receiving treatment [37–39]. These exercises must be individualized but often should include isometric (static) strengthening, low-impact lower extremity strengthening, low-impact aerobic training (i.e., swimming or bike riding vs. running on a hard surface, and resistance training that does not generate pain nor put stress on the spine [40]. A recent trial has found feasibility of individually adapted free exercises in stable and unstable spinal metastases and improved patient confidence in engaging in physical activity again; however, the study was limited by a small sample size and no effect on pain, QoL metrics, or survival [41]. Ideally, a cancer rehabilitation program would integrate these therapies in the period of pain relief that is provided by pharmacological measures and interventional approaches to maximize and restore patient functionality.

## Conclusion

Rehabilitation professionals and physicians who perform interventional pain procedures are often able to apply the same interventions they would for patients with LBP and no history of cancer. Doing so requires careful considerations of patient history, physical exam findings, tumor location, medications, which may negatively interact with interventional procedures (e.g., bevacizumab) and laboratory parameters (e.g., platelet level). In order for cancer rehabilitation to have an expanded role in improving pain and QoL for patients experiencing pain and who have VMD, there must be large-scale studies, demonstrating the safety and value of rehabilitation interventions on pain and quality of life in this population.

## Author Contributions

The figures were created by KS, EJ, and SS. All the authors participated in conceptualization of the manuscript and contributed to the writing and the revisions of the manuscript.

## Conflict of Interest

The authors declare that the research was conducted in the absence of any commercial or financial relationships that could be construed as a potential conflict of interest.
